# Mpox: An Overview of Pathogenesis, Diagnosis, and Public Health Implications

**DOI:** 10.3390/jcm13082234

**Published:** 2024-04-12

**Authors:** Francesco Branda, Chiara Romano, Massimo Ciccozzi, Marta Giovanetti, Fabio Scarpa, Alessandra Ciccozzi, Antonello Maruotti

**Affiliations:** 1 Unit of Medical Statistics and Molecular Epidemiology, Università Campus Bio-Medico di Roma, 00128 Rome, Italy; f.branda@unicampus.it (F.B.);; 2 Sciences and Technologies for Sustainable Development and One Health, Università Campus Bio-Medico di Roma, 00128 Roma, Italy; 3 Climate Amplified Diseases and Epidemics (CLIMADE), Brasilia 70070-130, Brazil; 4 Instituto Rene Rachou, Fundação Oswaldo Cruz, Belo Horizonte 30190-002, Brazil; 5 Department of Biomedical Sciences, University of Sassari, Viale San Pietro 43b, 07100 Sassari, Italy; 6 Department GEPLI, Libera Università Maria Ss Assunta, 00193 Rome, Italy

**Keywords:** Mpox, epidemiology, surveillance, Orthopoxvirus

## Abstract

Mpox, caused by viruses of the genus Orthopoxvirus, is an emerging threat to human and animal health. With increasing urbanization and more frequent interaction between humans and wild animals, the risk of Mpox transmission to humans has increased significantly. This review aims to examine in depth the epidemiology, pathogenesis, and diagnosis of Mpox, with a special focus on recent discoveries and advances in understanding the disease. Molecular mechanisms involved in viral replication will be examined, as well as risk factors associated with interspecific transmission and spread of the disease in human populations. Currently available diagnostic methods will also be discussed, with a critical analysis of their limitations and possible future directions for improving the accuracy and timeliness of diagnosis. Finally, this review will explore the public health implications associated with Mpox, emphasizing the importance of epidemiological surveillance, vaccination, and emergency preparedness to prevent and manage possible outbreaks. Understanding the epidemiology and control strategies for Mpox is critical to protecting the health of human and animal communities and mitigating the risk of interspecific transmission and spread of the disease.

## 1. Introduction

Monkeypox (Mpox) is a zoonotic infectious disease caused by the monkeypox virus (MPXV) and was first identified in 1970 in rural villages in the rainforest areas of Central and West Africa, when smallpox was in its final stages of eradication. MPXV is a double-stranded DNA virus belonging to the Poxviridae family, genus Orthopoxvirus (OPXV) (same genus as the variola virus that causes smallpox and the vaccinia virus that causes cowpox). There are two genetically distinct clades of MPXV: Clade I (formerly the Central African, Congo Basin clade) and Clade II (formerly the West African clade). Clade II is further subdivided into Clade IIa and IIb. Clade I is clinically more severe, with greater interhuman transmissibility and greater lethality. MPXV is widespread in particular among primates and small rodents, mainly in Africa. This includes squirrels, Gambian marsupial rats, dormice, non-human primates, and other species. However, uncertainties remain about what the virus’s natural reservoirs are and how its circulation in nature is maintained. Mpox is endemic in Central and West Africa, where outbreaks are regularly reported, especially in the Democratic Republic of Congo (DRC, formerly Zaire). In endemic countries, transmission occurs mainly from animals to humans. Until 2022, the rare cases that were reported in non-endemic countries were usually imported, while human-to-human transmission covered a limited percentage of cases. Mpox transmission in endemic countries occurs mainly from animals to humans through direct contact with infected animals, often during hunting, capture, and processing of infected animals or parts of infected animals and their fluids. Small mammals can carry the virus, sometimes without apparent symptoms, while non-human primates can get sick and have signs of the disease like humans. The natural reservoirs of the virus in endemic areas are not known. Person-to-person transmission occurs through close and prolonged contact with a symptomatic person. Close contact means prolonged face-to-face contact (such as talking, breathing, or droplets); skin-to-skin contact (through contact with skin lesions and during sexual intercourse); mouth-to-mouth contact; or mouth-to-skin contact, which could be another way of transmission. Additionally, MPXV can be transmitted via contaminated objects, such as sheets, towels, and clothing. Human-to-human transmission through close physical contact, including sexual activity, is a significant factor that can trigger an epidemic event.

## 2. Epidemiological Surveillance: Data Availability, Management, and Modeling

The initial stages of an infectious disease outbreak resemble a small brush fire. The containment of such outbreaks relies on swiftly extinguishing chains of transmission before they escalate into widespread epidemics. To be of any help, epidemiological modeling should offer short-term forecasts of Mpox incidence, addressing pertinent queries such as “Time to worry or not?” and “Could it be another threat?” [1]. Analyses must be robust, avoiding misleading inference due to strong and unreliable modeling assumptions [2,3], and thus must employ statistical methodologies aligned with surveillance conditions and be designed to address data quality and management challenges for enhanced modeling efficacy [4]. This is particularly true for the Mpox case. Indeed, the Mpox outbreak underscored the pivotal role of data collection, management, and accessibility in statistical approaches for policy formulation, drawing essential lessons from the challenges encountered during the COVID-19 pandemic. Like the COVID-19 scenario, publicly available data for Mpox were frequently limited and inconsistent, particularly in the initial phase of the outbreak, and unfortunately, this issue persists. Access to more accurate information was impeded by challenges in data collection, ambiguous privacy policies, and institutional reluctance to share data, thereby impeding a comprehensive understanding of the dynamics of the Mpox outbreak [5] and hindering the implementation of effective, data-driven policy-making [6]. Thus, modeling endeavors should be largely directed toward managing unreliable, temporally and spatially misaligned, incoherent, and incomplete data related to Mpox. Addressing data quality issues necessitated the development of strategies that acknowledge relevant uncertainties in the estimation process. The inferior quality of the data posed challenges to the application of highly sophisticated statistical models, which ideally require timely and high-quality data to yield accurate and reliable results. To achieve this, data-driven approaches, selecting a suitable parametric count distribution and a logistic-type time trend for the available aggregated data, should be preferred for, e.g., compartmental models [7]. To facilitate comprehensive research, anonymized data from real-time surveillance systems, designed with privacy safeguards, should be made openly accessible to all researchers. Such an approach aligns with the principles of the open-data community, fostering the rapid scientific progress crucial in addressing public health crises like the Mpox outbreak. Good-quality data feed reliable models, which provide outputs useful to plan policy interventions. Efforts should be directed towards evaluating the impact of both pharmaceutical interventions (PIs) and non-pharmaceutical interventions (NPIs). Nevertheless, most analyses during this period adopted a rather simple and often spurious correlational approach, with a few exceptions. It is essential to caution against relying solely on correlational studies for decision-making, as they pose the risk of basing decisions on coincidental relationships. Conversely, conducting rigorous causal studies to assess the effectiveness of pharmaceutical and non-pharmaceutical interventions demands disaggregated or individual data for estimating counterfactual models. Unfortunately, such granular data were not readily available during the initial stages of the pandemic.

## 3. Molecular Aspects of Viral Replication and Immune Response

### 3.1. Molecular Mechanisms of Viral Replication

Viral replication represents a complex and highly orchestrated process involving a series of molecular events aimed at hijacking the host cellular machinery to facilitate the production of viral progeny [8]. Upon successful entry into the host cell, which is facilitated by specific interactions between viral surface proteins and cellular receptors, the MPXV genome is uncoated and released into the cytoplasm [9]. This genome, composed of linear double-stranded DNA, encodes numerous genes responsible for various stages of viral replication. One of the key steps in viral replication is the initiation of viral DNA synthesis, mediated by the viral DNA polymerase complex. This enzyme complex utilizes host cell nucleotides to synthesize complementary DNA strands, ultimately leading to the production of full-length viral genomes [10]. Concurrently, viral mRNA transcripts are generated through the process of transcription, mediated by viral RNA polymerases. These transcripts serve as templates for the translation of viral proteins essential for replication, including enzymes involved in DNA synthesis, such as DNA helicases and DNA primases, as well as structural proteins required for the assembly of new viral particles. As the infection progresses, a cascade of late gene expression occurs, leading to the synthesis of additional viral proteins necessary for the assembly and maturation of virions. This includes structural proteins such as the major capsid protein, membrane proteins, and proteins involved in virion assembly and morphogenesis. The orchestrated expression of these late genes ensures the efficient production of infectious viral particles. Once assembled, mature virions are transported to the cell periphery and released from the infected cell, ready to infect neighboring cells and propagate the infection. Notably, the process of viral replication is tightly regulated and coordinated, with viral proteins interacting with host cell factors to modulate cellular processes and favor viral replication. Additionally, viral replication occurs in discrete intracellular compartments known as viral factories, where viral proteins and nucleic acids are concentrated to facilitate efficient replication and assembly. Understanding the molecular mechanisms underlying viral replication is essential for the development of targeted antiviral therapies and vaccines against MPXV and related orthopoxviruses (OPXVs). By elucidating the intricate interactions between the virus and the host cell during replication, researchers can identify novel targets for therapeutic intervention and devise strategies to disrupt viral replication and prevent the spread of infection. Moreover, insights into the molecular basis of viral replication can inform the design of attenuated or vectored vaccines capable of eliciting robust immune responses against MPXV, thereby providing long-term protection against infection [11]. Continued research into the molecular virology of MPXV replication will be crucial for advancing our understanding of virus–host interactions and developing effective strategies to combat this emerging infectious disease.

### 3.2. Molecular Mechanism of Host Immune Response

The host immune response orchestrates a multifaceted defense against MPXV infection, encompassing both innate and adaptive immune mechanisms [12]. Upon encountering viral components, innate immune cells including dendritic cells, macrophages, and natural killer cells are swiftly activated, triggering an inflammatory cascade. This response is characterized by the secretion of proinflammatory cytokines and chemokines such as tumor necrosis factor-alpha (TNF-α), interleukin-1 (IL-1), and interferons (IFNs), which recruit and activate other immune cells to the site of infection [13]. Dendritic cells, key sentinels of the immune system, play a pivotal role in initiating the adaptive immune response against MPXV [14]. They capture viral antigens and migrate to lymphoid organs where they present these antigens to T cells. This process leads to the activation and clonal expansion of antigen-specific T cells, including CD4+ helper T cells and CD8+ cytotoxic T cells. CD4+ T cells provide crucial help to B cells, facilitating their differentiation into antibody-secreting plasma cells and enhancing the production of neutralizing antibodies [15]. Meanwhile, CD8+ T cells directly target and eliminate infected cells through the release of cytotoxic granules containing perforin and granzymes, thereby limiting viral spread within the host. In addition to cellular immunity, humoral immunity mediated by B cells and antibodies constitutes a vital component of the host defense against MPXV. B cells undergo activation and differentiation into plasma cells upon encountering viral antigens, resulting in the production of virus-specific antibodies [16]. These antibodies can neutralize the virus by binding to viral surface proteins, preventing viral attachment and entry into host cells. Furthermore, antibodies can facilitate the clearance of viral particles by opsonization, marking them for phagocytosis by macrophages and neutrophils. The adaptive immune response mounted against MPXV infection also leads to the generation of immunological memory, conferring long-term protection against reinfection [12]. Memory T and B cells persist in the host following resolution of the acute infection, enabling a rapid and robust response upon re-exposure to the virus. Memory T cells exhibit enhanced effector functions and can quickly proliferate and differentiate into cytotoxic T cells upon encountering viral antigens, providing immediate protection against viral spread. Despite the host’s formidable immune response, MPXV has evolved various strategies to evade immune detection and establish persistent infection. The virus can modulate host cell signaling pathways involved in antigen presentation, impairing the recognition and elimination of infected cells by immune effectors. Additionally, MPXV encodes proteins that interfere with cytokine signaling pathways, dampening the host’s antiviral response and facilitating viral replication and dissemination [12]. Understanding the dynamic interplay between MPXV and the host immune system is paramount for the development of effective vaccines and therapeutics against MPXV infection. By elucidating the molecular mechanisms underlying immune evasion and identifying key targets for intervention, researchers can design strategies to bolster host immunity and curtail viral spread. Moreover, insights into the host immune response to MPXV infection can inform the development of next-generation vaccines capable of eliciting robust and durable immunity against this emerging infectious disease.

### 3.3. Evasion Strategies of the Virus

**Figure 1:**
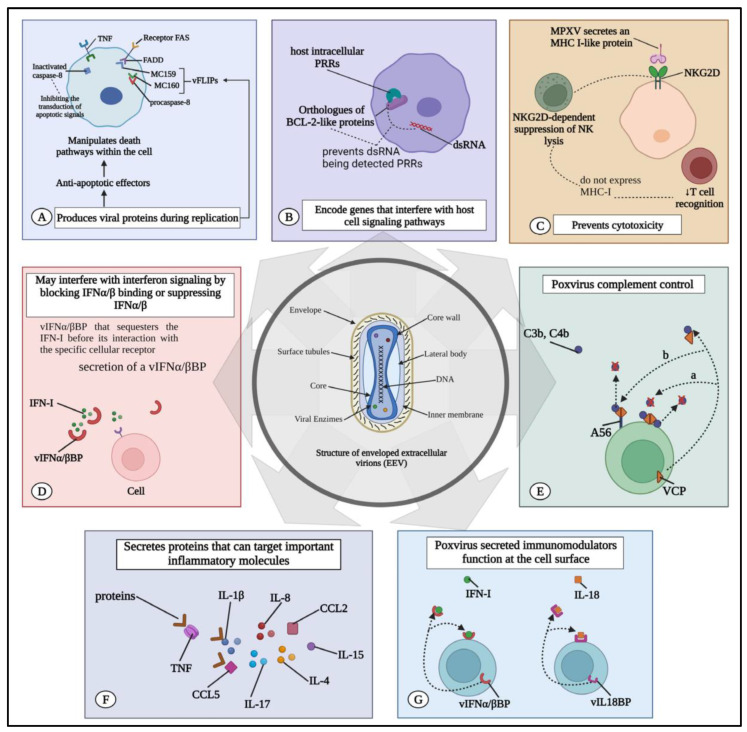
MPXV immune evasion mechanisms. (**A**) MPXV triggers the production of viral proteins that inhibit the host’s response. These proteins, which have antiapoptotic effects, can either be released to counteract signals from the external environment or manipulate cell death pathways within the cell itself. (**B**) The virus encodes proteins that counteract pattern recognition receptors (PRRs). For instance, MPXV’s BCL-2-like proteins prevent the detection of double-stranded RNA (dsRNA) by intracellular PRRs in the host. (**C**) It hinders T-cell-mediated and natural killer (NK) cell-mediated cytotoxicity. MPXV releases MHC-class-I-like protein (MHC-I) that binds to NKG2D, thereby inhibiting the typical NKG2D-dependent NK cell lysis of infected cells lacking MHC I expression and consequently reducing T cell recognition. (**D**) MPXV may interfere with interferon signaling by either blocking IFN-α/β binding or suppressing IFN-α/β production. For instance, it produces vIFN-α/β binding protein (vIFN-α/βBP) that sequesters IFN-I before it can interact with specific cellular receptors. (**E**) The poxvirus complement control protein acts not only as a decoy receptor for cytokines but also as a secreted immunomodulator. It binds to C3b and C4b in solution and can attach to the cell surface. (**F**) MPXV releases proteins that target key molecules such as IL-1β, IL-1 receptor antagonist (IL-1RA), IL-2 receptor (IL-2R), IL-4, IL-5, IL-6, IL-8, IL-13, IL-15, IL-17, CCL2, and CCL5. (**G**) It produces an IL-18 binding protein (vIL18BP) that further inhibits the cytotoxic activities of NK cells. vIL18BP suppresses IL-18-induced interferon production and a pattern of Th1 cytokines necessary for the expansion of cytotoxic T lymphocytes (CTLs) and NK cells. The image presented in this figure was sourced from Lucena-Neto et al. [22] and edited using the software GIMP 2.8 (available at https://www.gimp.org/downloads/oldstable/, accessed on 5 April 2024) in compliance with the terms of use for CC BY 4.0 DEED license (https://creativecommons.org/licenses/by/4.0/, accessed on 5 April 2024).

MPXV has evolved sophisticated strategies to evade immune detection and establish persistent infection within the host [10]. For details on the various mechanisms through which MPXV can evade the immune system, see Figure 1. These evasion tactics target various components of the host immune response, allowing the virus to subvert antiviral defenses and promote its survival and dissemination. One strategy employed by MPXV involves the modulation of host innate immune signaling pathways. Upon infection, the virus encodes a range of immunomodulatory proteins that interfere with the production and signaling of host antiviral cytokines, including interferons (IFNs) and proinflammatory cytokines [17]. For instance, MPXV produces soluble cytokine decoy receptors, which competitively bind to host cytokines, preventing their interaction with cellular receptors and dampening the immune response [18]. Additionally, the virus secretes proteins that disrupt downstream signaling pathways activated by cytokine receptor engagement, thereby inhibiting the expression of antiviral genes and promoting viral replication. Furthermore, MPXV can evade immune detection by modulating host cell signaling pathways involved in antigen presentation and recognition [19]. The virus interferes with major histocompatibility complex (MHC) class I antigen presentation, either by downregulating MHC class I expression on infected cells or by inhibiting the processing and presentation of viral antigens. This impairs the recognition of infected cells by cytotoxic T lymphocytes (CTLs), allowing MPXV to evade immune surveillance and establish persistent infection. Moreover, MPXV can inhibit the expression of co-stimulatory molecules on antigen-presenting cells, impairing T cell activation and proliferation in response to viral antigens [20]. Additionally, MPXV has evolved mechanisms to evade detection by the host adaptive immune system. The virus can undergo antigenic variation, generating diverse viral variants with altered antigenic profiles that evade recognition by neutralizing antibodies and T cells. This antigenic diversity enables MPXV to escape immune surveillance and persist in the host population. Furthermore, the virus can modulate the expression of viral antigens on the surface of infected cells, either by downregulating antigen presentation machinery or by masking antigenic epitopes with viral proteins. This reduces the susceptibility of infected cells to recognition and elimination by immune effectors, allowing MPXV to establish persistent infection within the host. Moreover, MPXV has developed mechanisms to evade host cell apoptosis, prolonging the survival of infected cells and facilitating viral replication and dissemination. The virus encodes proteins that inhibit apoptosis, either by interfering with the activation of proapoptotic signaling pathways or by directly blocking the execution of apoptotic cell death. This prolongs the lifespan of infected cells, allowing MPXV to replicate to high levels and spread to neighboring cells without triggering immune-mediated clearance. Overall, the evasion strategies employed by MPXV underscore the virus’s remarkable ability to subvert host immune defenses and establish persistent infection [21]. Understanding the molecular mechanisms underlying immune evasion is crucial for the development of effective countermeasures against MPXV infection. By elucidating the interplay between the virus and the host immune system, researchers can identify novel targets for therapeutic intervention and devise strategies to enhance host immunity and control viral spread. Moreover, insights into MPXV immune evasion mechanisms may inform the design of next-generation vaccines capable of eliciting robust and durable immune responses against this emerging infectious disease. Continued research into the immunopathogenesis of MPXV infection will be essential for advancing our understanding of virus–host interactions and improving clinical outcomes for infected individuals.

## 4. Diagnostic Methods for MPXV

### 4.1. Mpox Laboratory-Based Testing Methods

Identifying Mpox based on symptoms alone is challenging due to the similarity in clinical manifestations caused by different OPVs [23]. Early detection is crucial for identifying infected individuals, alerting them to take timely isolation and treatment measures, and thereby reducing the virus spread and mitigating the impact of an outbreak [24]. There is an increasing need to develop diagnostic techniques that are highly sensitive, accurate, and capable of fast detection rates. Specifically, the test should be able to distinctly detect MPXV, no other similar viruses, with high sensitivity to ensure accurate and reliable results. It should detect minuscule amounts of MPXV and be able to identify the virus in the early stages of infection for effective outbreak prevention [25]. After an Mpox outbreak, quickly diagnosing the disease and detecting the causative strain is necessary. Since different clades may have varying epidemiological characteristics, understanding these can help formulate relevant epidemic prevention measures and facilitate vaccine development. However, branch testing for specific Mpox clades should only occur after confirming the virus as MPXV to avoid false negatives due to the virus’s evolution and the emergence of new branches. Currently, polymerase chain reaction (PCR) remains the most common technique for MPXV testing, although other methods like immunological assays and virus isolation from cell culture are also available [26]. This article reviews current laboratory techniques for MPXV detection, discusses progress in method improvement, and compares the strengths and limitations of various diagnostic tests. It aims to assist medical professionals in selecting suitable assays for different contexts and proposes novel assays that could enhance diagnosis and facilitate the development of new diagnostic tools.

### 4.2. Nucleic Acid Amplification Testing (NAAT)

Nucleic acid amplification testing (NAAT) encompasses both real-time and conventional PCR techniques for the confirmation of MPXV infections. These methodologies are integral for the identification of unique viral DNA sequences that are characteristic of MPXV [27]. PCR, whether applied alone or in conjunction with sequencing, is essential for the delineation of viral clades, effectively differentiating between the Congo Basin (Clade I) and West African (Clade II) virus strains [28]. The formulation of validated PCR protocols has been the result of collective efforts across various research entities, yielding approaches that not only detect OPXV but also specifically identify MPXV, facilitating the distinction of its clades. The academic community has contributed extensively to this field, providing a plethora of primer and probe sequence sets that enable the development of in-house assays in well-equipped laboratories. Certain protocols employ a bifurcated strategy where an initial PCR reaction discerns the presence of OPXV without specifying the species, followed by a secondary PCR-based or sequencing effort to precisely identify MPXV clades or lineages [26]. The emergence of commercial PCR test kits targeting OPXV and MPXV has been noted, with performance evaluations underscoring those exhibiting superior sensitivity and specificity. The incorporation of positive control material, procured through specialized initiatives, is advocated to affirm the assay’s analytical performance. Implementing appropriate controls is paramount in preserving specimen and assay integrity, thereby mitigating false negatives and fortifying the reliability of the diagnostic process.

### 4.3. Electron Microscopy

Electron microscopy offers a technique for the visual identification of potential poxviruses within specimens. However, due to the need for specific technical expertise, requisite facilities, and the proliferation of more accessible molecular assays, its application in MPXV diagnosis is not widespread [29,30]. Consequently, electron microscopy is infrequently utilized in the routine diagnostic assessment of poxviruses.

### 4.4. Virus Isolation and Culture

Virus isolation and culture are time-honored methods integral to diagnosing viral diseases, including MPXV. This process is crucial for in-depth characterization through sequencing, enabling the testing of antivirals, vaccine development, and the formulation of clinical applications and research methodologies [31]. Isolating viruses from key cases aids outbreak investigation and containment efforts by identifying the virus’s origin, pinpointing mutations, and reconstructing transmission chains through genomic and phenotypic comparisons among isolates. MPXV demonstrates robust growth in mammalian cell lines such as HeLa, Vero, BSC-1, and RK-13, as well as in chicken embryos, which are notably susceptible to poxviruses [32]. The virus induces cytopathic effects in the chorioallantois membranes (CAMs) of chicken embryos, evident 1–4 days post-inoculation, including cell rounding, granulation, cytoplasmic bridging, and syncytium formation [33]. Conversely, when cultured in Vero cells, typical rounded and detached cells become observable within approximately 24 h, allowing for virus particle identification through immunofluorescence and specific antibodies. Despite its accuracy, this method’s extensive detection timeframe, the requirement for high-level biosafety labs (level 3 or higher), and the need for skilled personnel, alongside the risk of infection even with full personal protection, significantly constrain its widespread application.

### 4.5. Serology

The use of serology for the clinical diagnosis of MPXV, particularly via the detection of antibodies in plasma or serum, is advised against when conducted in isolation [34]. The efficacy of MPXV-specific serological tests is diminished by the potential for cross-reactivity with antibodies against other orthopoxviruses and those elicited by vaccination, whether recent or historical. It is suggested that serological testing be confined to reference laboratories until further substantiation is provided for the use of serological or antibody-detecting point-of-care (POC) tests outside such settings [17]. If a serologically validated test is available within a reference laboratory, the detection of IgM in recently acutely ill patients or IgG in paired serum specimens—collected at least 21 days apart from the initial sample obtained during the first week of illness—can augment diagnostic accuracy when other test results are inconclusive [35]. This approach emphasizes the nuanced role of serology in the diagnostic framework, underscoring its potential utility under specific conditions and within the confines of rigorous validation and application standards.

### 4.6. Whole-Genome Sequencing (WGS)

WGS, a pinnacle of next-generation sequencing technology, sequences an organism’s entire genome. It stands as the most precise method for differentiating MPXV from other orthopoxviruses (OPVs), offering expansive pathogen coverage beyond other molecular diagnostics [36]. WGS facilitates comprehensive bioinformatic analyses, promoting advanced virological study and the development of associated immunoassays. It excels in identifying specific strains and genetic variants, potentially elucidating outbreak origins, especially in cases of unknown transmission chains [37]. Additionally, WGS data can trace the virus’s genetic evolution, shedding light on its adaptation across diverse hosts and environments and identifying genetic markers for antiviral resistance or severe disease manifestations. Moreover, WGS enables the high-resolution mapping of MPXV phylogeny and biogeography, inferring virus migration patterns by comparing genome sequences from various outbreaks. Recognized increasingly as a vital tool in epidemiologic research, WGS’s contributions are instrumental in disease treatment, vaccine development, and the formulation of targeted outbreak prevention and control strategies. However, the technique’s demand for substantial computational resources, alongside high operational costs, limits its applicability to large-scale testing. The implementation of WGS faces challenges, including practical, ethical, and scientific considerations, requiring ongoing development and thoughtful policymaking [37]. While not suited for point-of-care testing due to these constraints, WGS’s insights are valuable for enhancing other diagnostic approaches. Its application primarily benefits research initiatives and specific case analyses, supported by the establishment of MPXV databases to consolidate findings.

## 5. Public Health Implications

Vaccination remains one of the most effective strategies to prevent and control Mpox outbreaks. The live-attenuated vaccine against Mpox, derived from vaccinia virus strains, has demonstrated its effectiveness in preventing severe disease and reducing transmission [38]. In endemic regions, targeted vaccination campaigns are essential to reach high-risk populations, including health workers, animal handlers, and individuals residing or traveling in affected areas [39]. In addition, routine immunization programs should be strengthened to ensure widespread vaccination coverage, particularly among children and adolescents [40]. Another crucial component is the early detection of Mpox cases, which is essential to initiate timely control measures and prevent further transmission. Strengthening surveillance systems, both nationally and internationally, is essential for early case identification and outbreak response [41]. Surveillance efforts should include syndromic surveillance, laboratory surveillance, and improved case-reporting mechanisms to facilitate rapid data collection and analysis [42]. In addition, the integration of One Health approaches that include animal health surveillance can provide early warning signals of potential spillover events from wildlife reservoirs to humans [43]. Moreover, strategic stockpiling and distribution of medical countermeasures are critical aspects of preparedness and response strategies for managing Mpox outbreaks. Adequate preparedness involves ensuring the availability of essential medical supplies, such as antiviral medications and personal protective equipment (PPE), to effectively mitigate the impact of outbreaks [44,45]. National and international collaboration is essential to ensure the timely procurement, stockpiling, and distribution of medical countermeasures. Health agencies at both levels should work together to assess the current stockpile levels, identify potential gaps, and coordinate procurement efforts to meet the anticipated demand during outbreaks. Furthermore, mechanisms for rapid deployment of medical supplies should be established to ensure swift response to outbreaks. This includes developing logistical plans for distribution, establishing communication channels for coordination among stakeholders, and implementing protocols for accessing and distributing medical countermeasures to affected areas [46]. Capacity-building efforts are also crucial to enhance preparedness and response capabilities. Healthcare workers should receive training on the proper use of PPE, including donning and doffing procedures, to minimize the risk of transmission during patient care [47]. Additionally, training programs should be implemented to ensure healthcare workers are proficient in administering vaccines and antiviral medications according to established guidelines. Community engagement and risk communication play pivotal roles in mitigating the impact of Mpox outbreaks by fostering awareness, building trust, and encouraging preventive behaviors within affected communities. Effective risk communication involves the dissemination of clear, culturally sensitive information about the signs and symptoms of Mpox, the significance of vaccination, and preventive measures like hand hygiene and avoiding contact with sick animals. Research indicates that tailored messaging delivered through various channels, including social media platforms [48] and news media [49], can effectively reach diverse populations and improve understanding of preventive measures. Public health authorities should collaborate with community leaders, religious institutions, and other stakeholders to develop and disseminate culturally appropriate educational materials and messages. Community-based organizations can serve as trusted sources of information and assist in reaching marginalized or hard-to-reach populations, thereby enhancing the effectiveness of risk communication efforts [50,51]. Furthermore, engaging with communities in a two-way dialogue fosters trust, addresses concerns, and promotes active participation in outbreak response efforts. Research suggests that involving community members in decision-making processes and soliciting feedback on intervention strategies can improve community acceptance and compliance with recommended measures [52,53]. Finally, international collaboration and coordination are indispensable components in managing Mpox outbreaks because of their propensity to spread across national borders. Such collaborative efforts are critical for pooling resources, sharing information, and coordinating response strategies among affected countries and international partners. Several key regional and global health organizations, including the World Health Organization (WHO) and the Africa Centres for Disease Control and Prevention (Africa CDC), serve as crucial facilitators in this regard. For example, during the 2018 Mpox outbreak in Nigeria, the WHO collaborated with national health authorities to conduct epidemiological surveys, deploy rapid response teams, and assist with case management and surveillance. International cooperation in response to Mpox outbreaks should prioritize several key areas, including:

1. Supporting outbreak response efforts: International partners can assist affected countries in deploying rapid response teams, establishing isolation and treatment facilities, and implementing control measures such as case detection, contact tracing, and vaccination campaigns [54].
2. Strengthening laboratory capacity: Enhancing laboratory diagnostics and surveillance capabilities is crucial for the timely detection and confirmation of Mpox cases. International collaboration can support the establishment of laboratory networks, training of personnel, and procurement of diagnostic reagents and equipment [55].
3. Conducting research: Research efforts should focus on advancing the understanding of Mpox epidemiology, transmission dynamics, and host–pathogen interactions. International collaboration enables the sharing of research findings, data analysis, and coordination of multicenter studies to address knowledge gaps and inform evidence-based interventions [56].

## 6. Discussion

Mpox is usually a self-limiting disease and typically lasts 2 to 4 weeks. Some people may develop more severe disease and require hospitalization. The prognosis of the disease depends on multiple factors including previous vaccination status, the person’s initial health status, concomitant diseases, and comorbidities. After an incubation period that can vary from 5 to 21 days, the disease is generally characterized by: a prodromal phase, which lasts between 0 and 5 days, with fever, intense headache, lymphadenopathy, myalgia, and intense asthenia. Lymphadenopathy is a distinctive feature of Mpox compared to other diseases that may initially appear similar (chickenpox), a rash that usually presents within 1–3 days of the onset of fever, typically starting on the face (involved in 95% of cases) and then spreading to other parts of the body, especially the extremities. The oral mucous membranes (in 70% of cases), the genitals (30% of cases), and the conjunctivae (20%) may also be involved. The rash generally progresses sequentially from macules to papules. The 2022–2023 Mpox outbreak marked a significant deviation from previous patterns of transmission, clinical presentation, and affected populations, highlighting the evolving nature of infectious diseases in a globalized world. The outbreak, first identified in the United Kingdom on 6 May 2022, quickly spread across 35 European countries and territories by 13 July 2022 [57], underscoring the rapidity with which Mpox can disseminate beyond traditionally endemic regions. A notable aspect of this outbreak was the change in the clinical picture of Mpox [58]. Historically, Mpox has predominantly affected children in endemic regions. However, the recent epidemic has shown a significant demographic shift, with most cases now occurring in adults. This change is partly attributed to transmission patterns and perhaps changes in immunity within the population over time. In particular, the epidemic has been particularly prevalent among men who have sex with men (MSM), suggesting new dynamics in the spread of the virus. The recent outbreak also observed changes in the typical distribution of the rash, with more frequent lesions on the face, genitalia, and mucous membranes. This differs from previous observations, in which the rash distribution was more widespread throughout the body. The concentration of lesions in more localized areas, including sensitive regions, underscores the need for healthcare providers to be alert and aware of these changes to make accurate diagnoses. Prodromal symptoms, which occur before the appearance of the characteristic rash, have also evolved. Patients often report experiencing a prodromal phase that includes fever, intense headache, lymphadenopathy (swelling of lymph nodes), myalgia (muscle pain), and intense asthenia (weakness). Lymphadenopathy, in particular, has been noted as a distinguishing feature of Mpox compared with similar diseases, such as chickenpox, and its presence may be a key differentiator in clinical diagnosis. A study conducted in an Italian research hospital [59] during the 2022 outbreak provided insights into the clinical presentation of Mpox. It showed mostly mild symptoms with complete resolution after 7–10 days without the need for antiviral therapy. This outbreak showed a shift in transmission routes, particularly through unprotected sexual intercourse, with most lesions concentrated in the genital and perianal areas. Interestingly, subjects vaccinated against smallpox showed milder symptoms, suggesting a protective effect.

Our analysis tries to remark on the objectivity of an infectious disease mainly due to travel in notoriously endemic areas, a disease which can then be transmitted in different ways, including predominantly sexual ones without protection, regardless of sexual orientation. Our article emphasized on the need to inform people traveling in areas at risk to resort to vaccination, which exists and is available, moreover, at a territorial level widespread information on this disease and surveillance are important. In light of the evolving epidemiology of Mpox, as evidenced by the 2022–2023 outbreak, our work reiterates the need for continued research, surveillance, and adaptive public health interventions to effectively address this evolving threat. The knowledge gained from recent outbreaks should inform future strategies to mitigate the spread of Mpox, underscoring the importance of a coordinated international response to emerging infectious diseases. Moreover, the recent outbreak also emphasizes the critical role of vaccination and public health awareness in controlling the spread of Mpox. With vaccines available, efforts must be intensified to promote vaccination among high-risk groups, especially individuals traveling to endemic areas. Public health campaigns should focus on raising awareness about the modes of Mpox transmission, emphasizing the importance of preventive measures including vaccination, safe sexual practices, and personal hygiene.

## Data Availability

No new data were created or analyzed in this study. Data sharing is not applicable to this article.
